# Serological field investigations revealing natural exposure of one-humped dromedary camels (*Camelus dromedarius*) to foot-and-mouth disease virus in India

**DOI:** 10.3389/fcimb.2025.1615461

**Published:** 2025-08-06

**Authors:** Manoranjan Rout, Lenin Bhatt, Jajati Keshari Mohapatra, Saravanan Subramaniam, Rabindra Prasad Singh

**Affiliations:** ^1^ ICAR-National Institute on Foot and Mouth Disease, International Centre for Foot and Mouth Disease, Bhubaneswar, India; ^2^ State FMD Unit, Jaipur, Rajasthan, India

**Keywords:** dromedary camel, foot-and-mouth disease virus, INDIA, nonstructural protein antibodies, Rajasthan

## Abstract

Foot-and-mouth disease (FMD) is one of the most significant animal diseases globally, affecting over 60 susceptible species including camelids particularly Bactrian camels. In order to gather baseline evidence on the current status of FMD in Indian camels, a preliminary random serosurvey was conducted in camels of Rajasthan state with significant camel population. A total of 777 sera collected from one-humped dromedary camels (*Camelus dromedarius*) across 11 districts of Rajasthan during 2016–2017 were screened for FMD virus (FMDV) 3ABC nonstructural protein (NSP)-antibodies using the commercial PrioCHECK^®^ FMDV NS kit. The sera were further tested using in-house liquid phase blocking (LPB) ELISA to evaluate the level of protective structural protein (SP)-antibody responses against three vaccine strains of FMDV serotypes O, A and Asia 1 if any. Out of 777 serum samples, 117 (15.05%; 95% Confidence Interval: 12.6%-17.7%) tested positive for 3ABC NSP-antibodies indicating the exposure of camels to FMDV. However, none of the sera tested in LPB ELISA showed a protective log_10_ antibody titre of ≥1.8 against any of the three FMDV serotypes confirming the absence of vaccination in these animals. This report provides retrospective evidence of anti-FMDV antibody response in dromedaries in India. Nevertheless, the role of dromedaries in epidemiology and transmission of FMDV remains unclear, emphasizing the need for further extensive serological screening of a larger number of samples. To the best of our knowledge, the present study, although preliminary, seems to be the first of its kind reporting the evidence of anti-FMDV antibody response in dromedaries in India.

## Introduction

Camel is an even-toed ungulate in the genus *Camelus*, which belongs to the suborder *Tylopoda* within the order *Artiodactyla* (Wernery and Kaaden, 2002). There are three existing species of camel such as, the one-humped dromedary camel (*Camelus dromedarius*) constituting 94% of the world’s camel population, the two-humped Bactrian camel (*C. bactrianus*) and the wild Bactrian camel that is now critically endangered. According to the data available in the WRI Earth Watch database, the total global population of camels is 22 million, with over 15 million belonging to the dromedary species. In the arid desert landscape of the Rajasthan state in India, camel holds a special place as an icon and a crucial part of cultural identity of the desert communities. India holds 10^th^ position globally in terms of camel population, with a total camel population of 0.25 million (20^th^ Livestock Census All India Report, 2019). The camel population contributes around 0.04% of the total livestock population in India, where Rajasthan state leads in camel numbers with a population of about 0.21 million, followed by Gujarat (0.028 million), Haryana (0.005 million) and Uttar Pradesh (0.002 million). ICAR-National Bureau of Animal Genetic Resources (ICAR-NBAGR) declares 9 registered camel breeds in India, among which 5 breeds e.g., Bikaneri, Jaisalmeri, Jalori, Marwari and Mewari solely belong to the home tract of Rajasthan.

The Indian agrarian economy faces significant losses due to foot-and-mouth disease (FMD), which is recognized as the most challenging animal viral disease to control having substantial economic implications for livestock farmers (Alexandersen and Mowat, 2005). The FMD virus (FMDV) structural protein (SP)-antibodies (Abs) are elicited in FMD vaccinated animals, while infected animals produce antibodies to both the structural and nonstructural proteins (NSPs) (Clavijo et al., 2004; Yousef et al., 2012). As far as the species-susceptibility is concerned, opinions vary widely on susceptibility of camels to FMD. Though the World Organization for Animal Health (WOAH) code chapter on FMD includes camelids as species susceptible to FMD like that of cattle, buffalo, sheep, goat and pig in their potential involvement in the disease epidemiology, the infection dynamics differ across these species (OIE/WOAH, 2023). Wernery and Kinne (2012) extensively reviewed FMD in camelids, while Wernery and Kaaden (2004) reviewed FMD in South American camelids, in dromedaries and Bactrians. Larska et al. (2009) demonstrated that FMDV infection occurs in Bactrian camels, but not in dromedaries, although reports regarding susceptibility of dromedaries to FMD remain conflicting and inconclusive. It seems that dromedaries can contract the disease after experimental infection and through close contact with FMDV-infected livestock without posing a risk to other susceptible animals. Given this controversy, further research on FMD epidemiology in camels including large-scale serological investigation seems to be logical. The dearth of information on FMD in camels in India built the foundation of sparking curiosity to undertake the current study aiming to gather preliminary serological evidence of natural exposure of the resident dromedary camel population to FMDV in the Rajasthan state by assessing the presence of NSP-Abs. Given India’s extensive FMD control program with the goal of disease elimination, such investigations in species other than the primarily FMD-susceptible ones become immensely important.

## Method

### Study area and sampling

The serosurvey was conducted in Rajasthan state of India that is the largest state by area (342,239 km^2^), representing 10.4% of the country’s total area. It is located on the north-western side and comprises most of the vast Thar Desert, also known as Rajasthan Desert or Great Indian Desert. The state shares border with the Pakistani provinces of Punjab to the north-west and Sindh to the west, along the Sutlej-Indus River valley. Additionally, it is bordered by five other Indian states namely, Punjab to the north; Haryana and Uttar Pradesh to the north-east; Madhya Pradesh to the south-east and Gujarat to the south-west.

During the study, a simple questionnaire was formulated and used to gather information from farmers and herdsmen with data on the host and management practices. The herds were selected based on their history of being co-housed or co-reared with cattle and/or other ruminants like buffalo, sheep, goat, a common practice found among most of the herdsmen. At the same time, herds that were not co-housed with cattle but had an access to the shared grazing areas were also included in the selection process. Data on sampled animals, including species, age, sex, and breed were recorded along with the history of previous occurrences of FMD in the herd and FMD vaccination practice if followed. It is to note that some of the herdsmen were unable to declare the actual age of their animals during the survey. Further, the data collected revealed that FMD was well known to some herdsmen and they were aware of the disease, its clinical signs, seasonality and transmission, while some were not. A total of 777 serum samples were collected randomly from apparently healthy dromedary camels, aged between 1 and 12 years with a median age of 8 years. The samples were obtained from different breeds (Bikaneri, Jaisalmeri and Marwari) across 11 districts (Bikaner, Jodhpur, Pali, Jaisalmer, Ajmer, Udaipur, Bharatpur, Sawai Madhopur, Karauli, Jaipur, Alwar) of Rajasthan based on animal population density.

The sampling was conducted during the summer or pre-monsoon season (May to July) of 2016. Sampling sites were selected based on animal population density, specifically targeting locations where animals were kept in groups or reared individually by the owner. Door-to-door sampling was not performed. On pre-scheduled date, the selected locations were contacted through the village head along with the local veterinarians. Routine health check-up camps were organized during the morning hours, where the farmers were informed in advance and they brought their animals to the designated locations. During these health check-ups, blood samples were drawn from the jugular vein of the animal after proper restraint following all necessary hygienic and precautionary measures. The sampled camels showed no evidence of active clinical signs of FMD; even though they had unrestricted contacts while grazing together with other susceptible ruminants (cattle, buffalo, sheep and goats) and free-ranging wild herbivores in the region. Whole blood was collected in properly labeled vacutainers with clot activator and allowed to clot at ambient temperature for about 1 hour and transported to the laboratory. The serum was harvested after centrifugation at 4000 rpm for 15 minutes and transferred into labeled cryovials for storage at -40°C till serological analysis.

### Serological assays

### Nonstructural protein (NSP)-antibody detection assay

The PrioCHECK^®^ FMDV NS commercial ELISA kit (Prionics AG, Switzerland, Product No. 7610440) was used for FMDV 3ABC NSP-Ab detection as per the protocol. The kit follows a single dilution competitive or blocking ELISA, where the test plates were coated with 3ABC specific monoclonal antibody (mAb) followed by incubation with antigen (3ABC protein). The test was performed along with the supplied controls (negative, weak positive and positive control in duplicate as recommended) and the test samples @ 1:5 dilutions to individual wells of the plate. After overnight (16–18 hours) incubation at 22 ± 3°C, six continuous washes were given followed by addition of the ready-to-use conjugate [mAb horseradish peroxidase (mAb-HRPO)]. After incubation for 60 ± 5 minutes at 22 ± 3°C, the plate was washed six times as earlier without any holding time and the chromogen (TMB) substrate was dispensed to all wells. After incubation at room temperature (22 ± 3°C) for 20 minutes, the reaction was stopped with stop solution. After gentle mixing the well contents, color development in the form of optical density (OD) was measured at a wavelength of 450 nm using Tecan’s Sunrise absorbance microplate reader. For the test validity, the recommended criteria are that the OD_450_ max (mean OD_450_ of the negative control) must be >1.000, mean percentage inhibition (PI) of the weak positive control must be >50%, and the mean PI of the positive control must be >70%. The results were expressed as PI of the controls and the test sera using the formula:


$$\text{PI}\;=\;100\;-\;\left({\text{OD}}_{450}{\text{test}\;\text{sample/OD}}_{450}\text{negative}\;\text{control}\right)\times 100$$

Sera with PI ≥ 50% were scored as positive for antibodies against FMDV NSP (Sorensen et al., 1998).

### Vaccine-induced protective antibody detection assay

The randomly sampled camels were not vaccinated against FMD, as informed by the owners and local veterinarians, and it was even not a common practice to vaccinate these species against FMD in the region. However, to substantiate further, the serum samples were subjected to liquid phase blocking (LPB) ELISA to assess the level of protective SP-Ab response against all three component strains of FMDV serotypes O, A and Asia 1 in the inactivated trivalent vaccine. For this, two-fold dilution (from 1:16 to 1:128) of camel sera were tested using the in-house LPB ELISA kit (PDFMD, Mukteswar) as per the procedure described earlier (Ranabijuli et al., 2010). The results were expressed as percentage reactivity for each serum dilution as follows:


$$\text{Percentage}\;\text{reactivity}\;=\;\left({\text{OD}}_{\text{mean}\;\text{of}\;\text{each}\;\text{test}\;\text{serum}\;\text{dilution}}{\text{/OD}}_{\text{mean}\;\text{of}\;\text{antigen}\;\text{control}}\right)\;\times \;100$$

The antibody titres were expressed as logarithm of reciprocal of serum dilutions giving 50% of the absorbance recorded in the antigen control wells. The serum samples showing log_10_ titre of ≥1.8 were considered to have sufficient protective antibody (Sharma et al., 2015).

## Results

**Figure 1:**
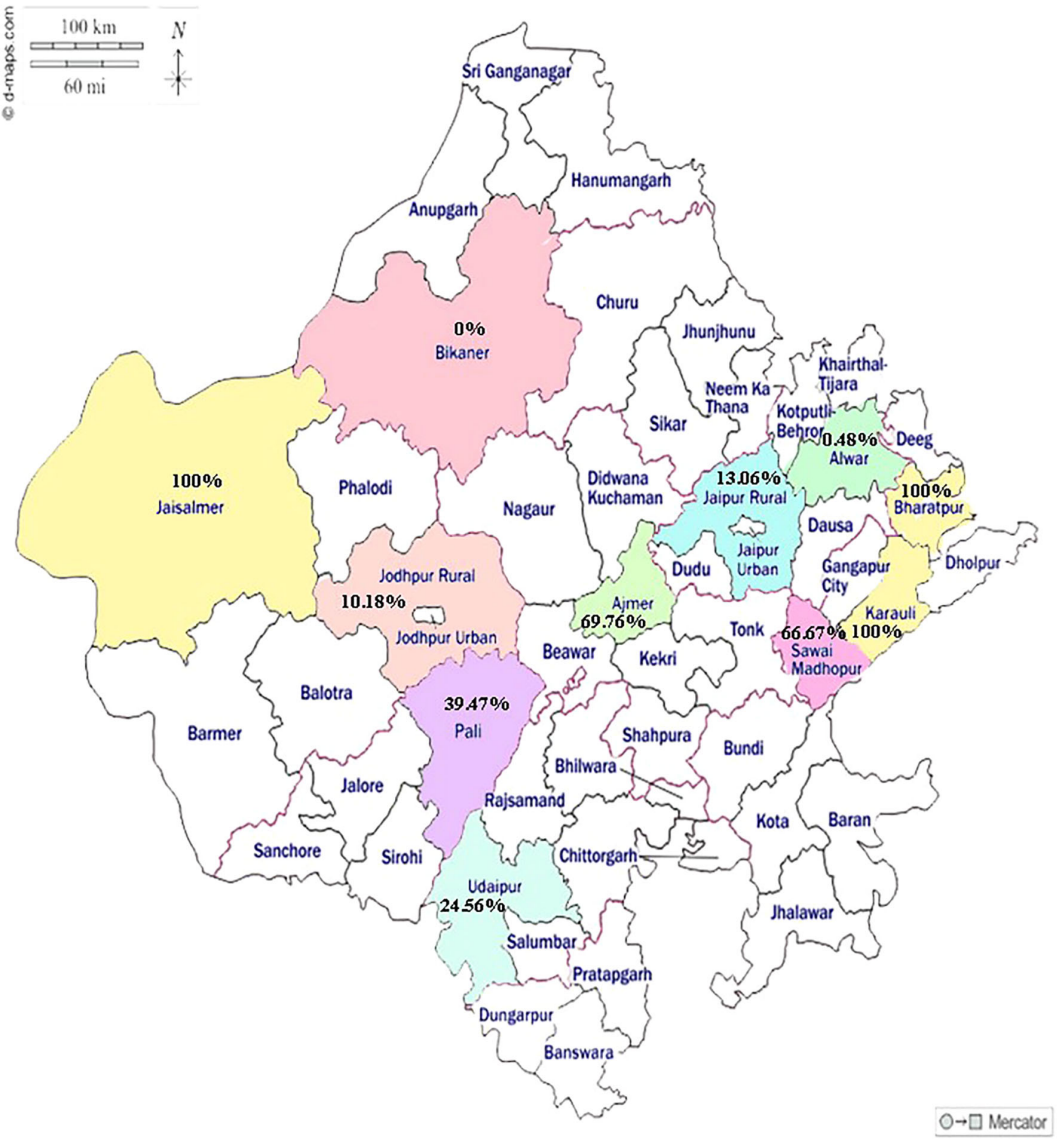
Map of Rajasthan state showing the prevalence percentage of NSP-Ab in camels in the sampled districts.

The NSP competitive ELISA detected 117 out of 777 serum samples (15.05%; 95% Confidence Interval: 12.6%-17.7%) as positive for anti-NSP antibodies. The district-wise number of camel serum samples tested and samples found positive against FMDV NSP-Ab along with their corresponding 95% confidence intervals have been illustrated in **Table 1**. The percentage apparent prevalence estimates across sampled districts followed a varied wider range of 0% (0/84) and 0.48% (1/207) in Bikaner and Alwar, respectively. The figure was 100% in three districts of Jaisalmer, Bharatpur and Karauli, where the sample size was of course very low (n ≤9), while 6 remaining districts showed percentage prevalence varying from 10% to 70%. The prevalence percentages of NSP-Ab in camels in the sampled districts have been depicted on the state map in **Figure 1**. However, none of the serum samples tested in LPB ELISA demonstrated protective log_10_ antibody titre of ≥1.8 against any of the three serotypes O, A and Asia 1.

**Table 1:** Table showing district-wise number of camel serum samples tested and samples found positive against FMDV NSP-Ab along with their corresponding 95% confidence intervals.

Name of the district	Number of animals sampled	No of seropositive (Percent positive)	95% Confidence Interval
Bikaner	84	0 (0%)	0.0-4.3
Jodhpur	108	11 (10.18%)	5.2-17.3
Pali	38	15 (39.47%)	24.0-56.6
Jaisalmer	9	9 (100%)	66.4-100
Ajmer	43	30 (69.76%)	53.9-82.8
Udaipur	57	14 (24.56%)	14.1-37.8
Bharatpur	3	3 (100%)	29.2-100
Sawai Madhopur	3	2 (66.67%)	9.4-99.2
Karauli	3	3 (100%)	29.2-100
Jaipur	222	29 (13.06%)	8.9-18.2
Alwar	207	1 (0.48%)	0.01-2.66
**Grand Total**	**777**	**117 (15.05%)**	**12.6-17.7**

## Discussion

FMD virus is a well-known and well-characterized contagion prevalent across the Asian continent, affecting both wild and domestic artiodactyls including the Bactrian camels. Results of experimental infection with the virus, and several field-based clinical observations spanning over a century confirm that the two closely related Bactrian and dromedary camels possess remarkably different susceptibilities to FMD. The present study depicting the NSP seropositivity rate of 15.05% indicates prior exposure of dromedary camels to FMDV and provides evidence supporting their ability to seroconvert against the virus. The LPB ELISA results confirm the absence of vaccination in camels in the region, as corroborated by information collected from the herdsmen and local veterinarians. Despite the evidence of seropositivity (El-Hakim, 2005; Yousef et al., 2012; Ularamu et al., 2015), the clinical susceptibility of dromedaries to FMD is not very apparent in the available literature (Wernery and Kinne, 2012).

Further, during the course of interaction with the farmers and herdsmen, it was evident that no FMD-like symptoms were observed in camels in past many years. Still, looking at the evidence of NSP-seropositivity in the sampled animals, it seems prudent to highlight some possible risk factors for probable exposure of camels to FMDV in the surveyed areas that could be co-housing of camels with other FMD-susceptible animals, free uncontrolled movement of camels with their frequent grazing access with other ruminants like cattle, buffalo, sheep, goat reared by many herdsmen under free-range conditions particularly at common grazing and water bodies. Additionally, camels are regularly moved across the deserts of Rajasthan due to seasonal variations, availability of grazing pasture, and for participation in fairs and trade activities, resulting in their intermingling with other susceptible animals as highlighted in regions, where FMD incidences occur in those species. Besides these risk factors, other facilitating risk-prone windows that can’t be ignored are absence of vaccination in camels, unregulated movement of both susceptible and infected animals, purchase of animals without following proper quarantine protocol, inadequate farm biosecurity measures and the proximity of farms to risk-prone areas. Under such circumstances, the situation of FMD is expected to be further complicated especially in a ‘no vaccination’ scenario in the suggested camel herds. Therefore, robust surveillance is of paramount importance to ensure that appropriate preventive protocols are in-place and strictly implemented to mitigate these risks and effectively control the spread of the virus during disease incidence situations.

It was observed that areas with high tourists’ movement such as Jodhpur (10.18%), Jaipur (13.06%), Udaipur (24.56%) and Ajmer (69.76%) exhibited higher antibody prevalence (range of 10% - 70%) in camels compared to other districts. While it is important to acknowledge the potential role of humans and fomites in the spread of the virus, no definitive claims can be made linking the reported seropositivity in dromedaries from these districts directly due to human activity-mediated transmission. It has been documented that people can carry FMDV on their clothes, footwear, skin and transmit it to susceptible animals. Notably, veterinarians and other individuals were implicated in FMDV spread during the 1967–1968 outbreak in the UK, contributing to 10 of recorded 51 outbreaks (MAFF Report, 1969). Although it is known that contaminated individuals have played a role in initiating new outbreaks (Gibbens et al., 2001; Sutmoller et al., 2003), there is insufficient data to reliably quantify this risk. It has further been documented that people can carry FMDV in their nasal cavities, but its likelihood of leading to infection in susceptible animals without close and prolonged contact is negligible (Auty et al., 2019). Anyway, while speculative, the higher prevalence in the tourist-heavy districts might warrant further investigation into potential indirect human activity-mediated transmission routes in camels as well as in other FMD-susceptible livestock species. Notably, the apparent prevalence of FMDV 3AB3 NSP-Abs in bovine population of Rajasthan during the time of sampling (2016-2017) from camels was higher at 35.73% (Annual Report, DFMD, 2016-2017) suggesting probable exposure of camels from seropositive bovine population. Such probability was deciphered as the camels and the cattle were co-housed in the areas by the farmers, which might have facilitated the exposure of the dromedaries to FMDV from the infected or ailing cattle in close proximity through several modes.

Despite the prevailing endemic setup in the country, government-aided control strategies and campaigns primarily focus on vaccinating the bovine population, while largely neglecting small ruminants, pigs and camels. Consequently, camels without any vaccinal immunity may potentially contribute to the transmission of FMDV and may carry the virus over long distance even across the borders (Yousef et al., 2012). The moderate seroprevalence figure of 15.05% observed in the present study aligns with the findings of Alexandersen et al. (2008) and Wernery and Kaaden (2004), who reported low susceptibility of camels to FMDV. In FMD serological study of dromedary camels in Oman conducted during the contemporary period of 2016–2017 by Body et al. (2019), sera from 151 local dromedaries co-grazing with mixed species (cattle and small ruminants) with FMDV NSP-Ab positive status were collected and tested for FMDV NSP-Ab using three commercial tests and were found negative indicating that FMDV was not transmitted to dromedaries kept with FMDV NSP-Ab positive ruminants. Meanwhile, in some serological field surveys, researchers have detected antibodies in dromedaries grazing alongside cattle, buffalo, sheep, goats and other free-ranging wild herbivores that agrees with our probable explanation as cited before for the seropositivity of dromedaries in similar situation. Although no clinical evidence of FMD was observed in those dromedaries, many had reportedly daily contacts and mingling with infected ruminants (Hedger et al., 1980; Farag et al., 1998). Seroconversion has also been reported in dromedaries in Ethiopia and Egypt (Abou Zaid, 1991) corroborating the earlier findings by Moussa et al. (1987), which indicated their susceptibility to natural FMDV infection. It has been suggested that dromedaries may develop antibodies following FMDV exposure even in the absence of clinical manifestations; however, these antibodies are believed to persist only for a short period. Yousef et al. (2012) documented 6.3% dromedary camels from different regions of Riyadh and Al-Qassim Province in the Kingdom of Saudi Arabia as positive for 3ABC NSP-Abs using PrioCHECK^®^ FMDV NS kit. More recently, Al-Husseiny et al. (2020) demonstrated 10% (52/520) seropositivity of NSP-Ab in one-humped camels (*Camelus dromedarius*) in the Middle of Iraq using the same PrioCHECK^®^ FMDV NS kit. Al-Khatib et al. (2012) reported 13.76% (19/138) seropositivity of dromedaries at different middle regions of Al-Riyadh Province in the Kingdom of Saudi Arabia using an indirect ELISA-based CHEKIT FMD-3ABC kit (IDEXX Laboratories). Similarly, Ularamu et al. (2015) reported 10.83% (39/360) positivity rate for 3ABC NSP-Abs among camel sera collected from abattoirs of different geo-political zones of Nigeria using PrioCHECK^®^ FMDV NS kit.

These findings provide serological evidence of FMDV exposure in camels, which could be attributed to their movement through areas that experienced FMD outbreaks and potential contact with infected cattle and small ruminants. Collectively, these reports confirm the serological evidence of anti-FMDV Abs in dromedaries and their ability to seroconvert. To the best of our knowledge, the present study, although preliminary, generates evidence of anti-FMDV antibody response in dromedaries for the first time in India. In the present study, although seroconversion was observed, none of the camels exhibited clinical signs of FMD. Contradictory reports exist regarding the susceptibility, clinical disease and seroconversion of dromedaries following FMDV exposure. While some researchers have reported susceptibility to clinical disease, others have documented only serological evidence. The isolation of FMDV has been reported in intranasally infected dromedaries from oropharyngeal region (Moussa et al., 1979), ruptured vesicles and ulcers of the upper lips of dromedaries (Moussa et al., 1987) as well as from tongue/gum epithelium of randomly selected dromedaries (Kumar et al., 1983) suggesting that dromedaries are susceptible to natural FMD (Yousef et al., 2012). Based on these reports, it may be presumed that dromedaries can contract the disease although with less susceptibility and through very close contact with FMD-affected livestock without posing any risk of further transmission to susceptible animals (Wernery and Kaaden, 2004; Alexandersen et al., 2008). In contrast, the findings of Wernery and Kinne (2012) certainly and clearly indicate that Bactrian camels can contract the disease, while dromedaries are not susceptible to FMD and do not transmit infection, even when in close contact with susceptible animals.

Although preliminary evidence in seropositivity of camels to FMD is gathered in this study, the need for further research into the pathogenesis and epidemiology of FMD in dromedaries cannot be overemphasized. Certain limitations of the study need to be acknowledged, particularly regarding comprehensive investigations including the collection of oropharyngeal fluid followed by virus genome detection using sensitive nucleic acid-based diagnostics or virus isolation in a substantial number of animals are essential. Nevertheless, parallel serological assessment targeting more than one NSP is also needed in order to establish the significance of dromedaries in FMD epidemiology. As this was a first cross-sectional study of its kind in the country involving a large number of dromedaries, due to certain constraints, repeated longitudinal samplings were not possible from the same herd, which limited the understanding on the antibody dynamics in the population. Furthermore, oropharyngeal fluid could not be collected from the NSP-Ab positive animals, preventing the confirmation of their subclinical status for FMDV. Another limitation was the absence of demographic data (such as age, sex and breed for the entire set of samples), which did not allow determining their ability to evaluate their potential association with seropositivity. Additionally, the interpretation of seroprevalence in districts like Jaisalmer, Karauli and Bharatpur should be approached with caution, as these districts reported 100% seropositivity based on very small sample sizes (n ≤ 9), which may not be representative of the population.

## Conclusion

To conclude, the present serological findings in dromedaries provide baseline indication of FMDV activity in this species substantiating their ability to seroconvert against FMDV. However, their role in the natural epidemiology and transmission dynamics of FMD remains to be investigated. To address this knowledge gap, more camel sera from different states where camels are found, need to be screened for anti-FMDV antibodies. Additionally, any lesions suggestive of FMD in camels should be carefully monitored and promptly investigated using reliable diagnostic tools for virus detection and isolation. Such a comprehensive approach will help elucidate the real status of the disease in these species. Further longitudinal analysis of serum samples in subsequent years to determine reduction in NSP-Ab prevalence in camels coincident with declaration of the same in cattle and buffalo population need to be conducted. This would demonstrate not only progressive reduction in FMD virus burden in the state of Rajasthan, but could also suggest dynamics of FMDV transmission from cattle to camels.

## Data Availability

The original contributions presented in the study are included in the article/**Supplementary Material**. Further inquiries can be directed to the corresponding author.
