# Supplementary material for: Serological field investigations revealing natural exposure of one-humped dromedary camels (Camelus dromedarius) to foot-and-mouth disease virus in India
Source: Front Cell Infect Microbiol. 2025 Aug 6;15:1615461. doi: 10.3389/fcimb.2025.1615461 (PMC12364943; doi:10.3389/fcimb.2025.1615461)
Supplement: Supplementary file 1 [file DataSheet1.pdf]

Camel camp village ____________________

Date –

1.	Name of Owner	:	
2.	Address	:	
3.	Number of camels	:	
4.	Do you rear other livestock animals also except camels?	:	Yes / No
5.	If yes, then species and numbers	:	
6.	Feeding Practice	:	Stall Fed / Free Grazing
7.	Does animals of other owners also go for grazing with your animals?	:	Yes / No
8.	Does your animals come in contact with livestock of other owners while grazing?	:	Yes / No
9.	Does your animals come in contact with any wild animal while grazing?	:	Yes / No
10.	Have you purchased any animal from outside within last 6 months?	:	Yes / No
11.	How many Times do you go for health check-up of your animal in a Year	:	Yes / No
12.	Do you know about FMD and its signs in animals	:	Yes / No
13.	If yes, which signs?	:	
14.	Have you seen any of these signs in your camels?	:	Yes / No
15.	Have you seen any of these signs in your other animals?	:	Yes / No
16.	Have your animals been vaccinated for FMD?	:	Yes / No
17.	If yes, then date of last vaccination	:	
